# Acupuncture at Neiguan suppresses PVCs occurring post-myocardial infarction by alleviating inflammation and fibrosis

**DOI:** 10.1186/s13020-022-00606-7

**Published:** 2022-04-28

**Authors:** Hao Hong, Xin Cao, Tian Deng, Xiang-Min Meng, Yu-Meng Li, Li-Juan Zhu, Jing Lv, Xuan Li, Shu-Guang Yu, Bing-Mei Zhu

**Affiliations:** 1 Regenerative Medicine Research Center, West China Hospital, Sichuan University, Keyuan Road 4, Gaopeng Street, Chengdu, 610041 Sichuan China; 2 Acupuncture and Tuina School/Third Teaching Hospital, Chengdu University of Traditional Chinese Medicine, Shierqiao Road 37, Jinniu District, Chengdu, 610075 Sichuan China

**Keywords:** Post-myocardial infarction, Arrhythmia, Acupuncture, Autonomic nervous system, Inflammation

## Abstract

**Background:**

Acupuncture at Neiguan (PC6) has long been used for treating cardiovascular diseases, but its antiarrhythmic effect and the underlying mechanisms have not yet been well investigated, especially regarding premature ventricular complexes (PVCs) that occur post-myocardial infarction (MI). The purpose of this study was to study the antiarrhythmic effect of manual acupuncture applied to PC6 for a relatively long period (28 days) and to elucidate the mechanism in mice**.**

**Methods:**

An MI mouse model was generated by ligating the left anterior descending coronary artery in male C57/BL6 mice (n = 31). Manual acupuncture at PC6 was applied seven times weekly for 4 weeks. The state of myocardial injury was characterized by electrocardiography (ECG) and echocardiography. Inflammation was detected by ELISA and immunohistochemical stanning. Fibrosis was evaluated by Masson’s trichrome staining. RNA sequencing was used to explore the differentially expressed genes (DEGs) among the different groups after treatment.

**Results:**

Acupuncture at PC6 lowered the incidence of spontaneous PVCs after MI injury (1/9, 11%) compared to that in mice without acupuncture treatment (6/9, 67%) and improved the ejection fraction from 31.77% in the MI mice to 44.18% in the MI + PC6 mice. Fibrosis was reduced after PC6 treatment. RNA-seq showed many DEGs involved in the immune system and inflammatory response pathway. Further studies confirmed that inflammation at the circulation level and cardiac tissue was inhibited in MI + PC6 mice, accompanied by suppressed sympathetic activation.

**Conclusions:**

In conclusion, 28-day treatment of acupuncture at PC6 reduced spontaneous PVCs and improved systolic function, possibly by suppressing inflammatory response-mediated fibrosis and sympathetic hyperactivity.

##  Introduction

Premature ventricular complex (PVCs) is the most common ventricular arrhythmia after myocardial infarction (MI) and worsens the prognosis in patients with coronary heart disease [1]. Several studies have found that PVCs are associated with an increased risk of adverse cardiac events, particularly sustained ventricular arrhythmia and sudden death [2].

Inflammation and enhanced automaticity are considered as triggers of arrhythmias after myocardial infarction [3]. Inflammatory response comes up immediately after myocardial infarction, followed by activation of myofibroblasts that secrete matrix proteins in the infarcted area. Neutrophils and mononuclear cells are recruited to remove dead cells and matrix debris by phagocytosis, and then promote scar formation [4], contributing to cardiac fibrosis, so as providing the substrate for re-entry, which is closely related to the slow conduction of the surviving muscle tracts in the interstitial fibrosis region after myocardial ischaemia [5], and so that the arrhythmias. Inhibition of the inflammatory response is crucial for reducing fibrosis to improve heterogeneity and so that attenuate arrythmias [6, 7]. Enhanced automaticity induced by MI event is a common cause of PVCs [8, 9], and any approaches which inhibit sympathetic nerve system can reduce arrhythmias.

Currently, the treatment options for post-MI arrhythmias include antiarrhythmic drug therapy, defibrillation, and/or ablation. However, the administration of antiarrhythmic therapy in the months after MI has been discouraged because of the proarrhythmic risk [10]. The Cardiac Arrhythmia Suppression Trial (CAST) studied patients with ventricular arrhythmia after MI to determine whether anti-arrhythmic therapy could improve survival rates but unfortunately found that, despite the suppression of ectopy on Holter monitoring, patients treated with encainide, flecainide or moricizine had increased rates of sudden death and death from all causes [11].

Acupuncture, as a nonpharmacologic treatment against several diseases, including ischaemic heart disease, has been used for thousands of years to relieve angina and arrhythmias and improve cardiac function in clinical practice [12–14]. Numerous animal experiments have demonstrated the underlying mechanisms by which acupuncture can protect myocardial functions against ischaemia and ischaemia/reperfusion. Neiguan (PC6), one of the most commonly used acupoints in cardiovascular diseases, is located in the pericardial meridian and has been confirmed to attenuate symptoms of cardiovascular disease [15]. It has been demonstrated that EA treatment at PC6 for 5 days is effective for alleviating MI injury [16]. Our previous animal studies showed that acupuncture at PC6 both after and pre-MI treatment reduced myocardial infarction and ischaemia–reperfusion (I/R) injuries, respectively, and notably, an anti-arrhythmic effect of EA pre-treatment was also found after ischaemia–reperfusion (I/R) in rats [17, 18].

Although many studies have evaluated the immediate cardioprotective effects (including increased ejection fraction, modulated inflammation and reduced fibrosis) of acupuncture on myocardial infarction and I/R injury, the efficacy of acupuncture intervention against MI and arrhythmia was only observed within 7 days or less in those studies [16, 17, 19]. Few studies have been conducted on the suppression of ventricular arrhythmia induced by repeated acupuncture stimulation at PC6, and there is currently a lack of relevant molecular mechanisms. Thus, the goal of our present study was to evaluate the repeated stimulation effect of PC6 (28 days) on ventricular arrhythmia in an MI model of mice and to uncover the possible mechanism. We now focus on whether a 28-day repeated stimulation at PC6 could play anti-arrhythmic role by suppressing inflammatory response-mediated fibrosis and sympathetic hyperactivity.

## Materials and methods

### Experimental animals

Eight-week-old 20 ± 2 g C57BL/6 J male mice (n = 31) were supplied by Ensville Biological Technology Co., Ltd. (Chengdu, Sichuan, China). The mice were housed at a constant temperature (23 ± 1 °C) under a 12/12-h light–dark cycle with free access to food and water. All procedures were approved by the Ethics Committee for Animal Care and Use of Sichuan University, and all procedures were conducted in accordance with the guidelines of the National Institutes of Health Animal Care and Use Committee (No. 2020267A).

### Animal groups

Mice were randomly allocated to the sham-operated group (Sham, n = 13), the myocardial infarction group (MI group, n = 9), and the acupuncture group (MI + PC6 group, n = 9, Neiguan acupoint). The surgical procedures were performed as previously described [20, 21]. In brief, mice were anaesthetized by 3% isoflurane with 99.5% O_2_ and maintained under anaesthesia by 1–1.5% isoflurane. The left anterior descending (LAD) coronary artery was permanently ligated using a 7/0 monofilament suture (Shanghai Pudong Jinhuan Medical Instrument Co., Ltd.) to induce myocardial ischaemia. The ligation was confirmed by the pale appearance of the apex and anterior wall of the left ventricle. After the surgery, the mice were placed on a warm cushion until they were awake. Mice in the sham group were also subjected to the same procedure except for LAD ligation. The Lead II electrocardiogram was monitored before and after the operation.

### Acupuncture intervention

**Fig. 1:**
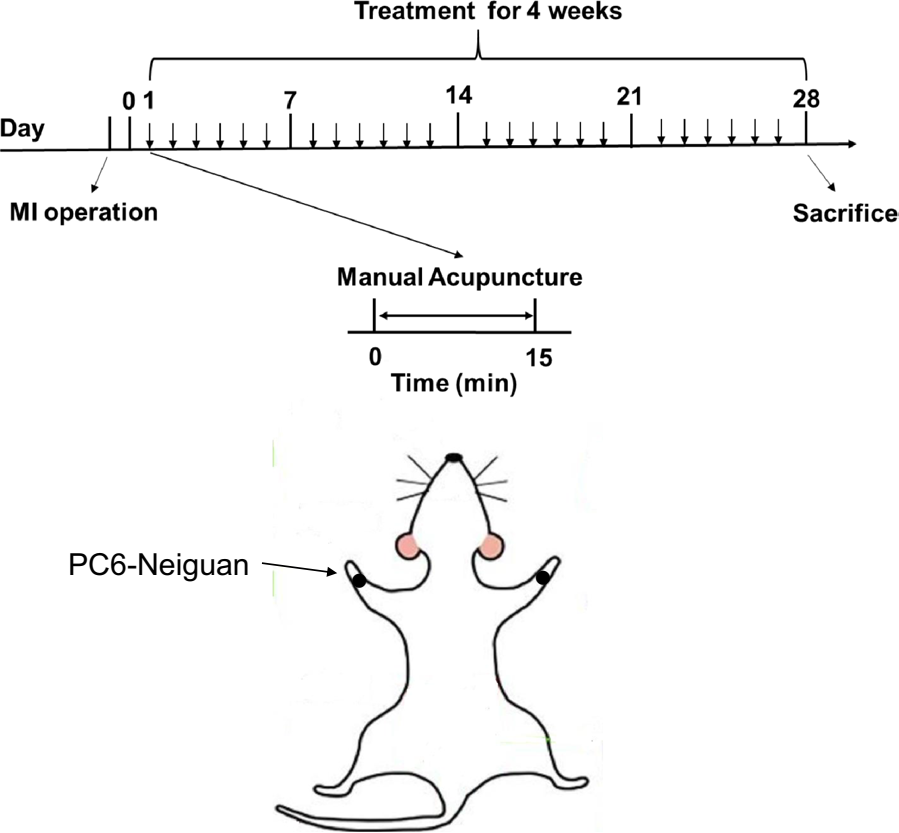
Timeline of the experiment and acupoint (Neiguan) location. The MI model was created by left anterior descending ligation, and 28 sessions of manual acupuncture were applied

The MI + PC6 group mice underwent acupuncture intervention awake at PC6 acupoint bilaterally after 24 h of MI operation once a day for 28 days. The acupuncture needles were folded into an “L” shape by hemostatic forceps and inserted into PC6 acupoint about 5 mm. Then the needles were fixed with adhesive tape. We also made sure that the needles would not fall off and strengthen the stimulation of acupuncture intervention every 5 min. The needles were removed after 15 min of intervention. Details for animal handling and fixation procedures are the same as our previous study [22]. PC6 was located at a point 1.5 cm proximal to the palm crease just above the median nerve (the experimental protocol and acupoint locations are depicted in Fig. 1).

### Electrocardiogram recording

Electrocardiograms were performed after 4 weeks of treatment. All mice were anaesthetized in the chamber at 3% isoflurane, carefully positioned on the electrocardiogram (ECG) recording platform and attached to a mask under 1.5% isoflurane. Surface lead II electrocardiogram was obtained. To minimize stress, we accomplished the electrode setup and system adjustment within 5 min, and thus, the first 5 min for each mouse were not included in our ultimate analysis. The next 5 min of ECG recordings were analysed by LabChart 8.2.3 (AD Instruments, Australia).

### Echocardiography analysis

After finishing the 28-day acupuncture treatment, all mice underwent transthoracic echocardiography under 1% isoflurane anaesthesia to characterize the effects of PC6 on cardiac structure and function using an ultrasound system (Vevo 3100, FUJIFILM Visual Sonics, Inc., Canada) equipped with an MX550D detector (25–55 MHz) of a wide-band frequency-fusion phase-array transducer. The heart was visualized in B mode from a long axis view. Left ventricle ejection fraction (EF) and fractional shortening (FS) were calculated from the measurements of wall thickness and chamber diameters. Left ventricle posterior wall thickness at diastole (LVPWd) and left ventricle anterior wall thickness at diastole (LVAWd) were measured in M-mode.

### Biochemical analyses

The serum levels of cTnT (JM-11710M1), TNF-α (JM-02415M1), IL6 (JM-02446M1), IL-10 (JM-02459M1), renin (JM-02627M1) and brain natriuretic peptide (BNP, JM-02343M1) were measured using commercially available kits (Jiangsu Jingmei Biological Technology Co. Ltd., China) in accordance with the manufacturer's instructions. All the methods and procedures strictly followed the protocols of the test kits.

### Haematoxylin and eosin staining

The ischemic heart tissues were dissected and then fixed with 4% paraformaldehyde immediately. Haematoxylin and eosin staining was performed on serial sections (4 μm thick) of paraffin-embedded heart tissues. Briefly, the sections were dewaxed in xylene, rehydrated in descending grades of ethanol and washed in distilled water. Excess water was blotted from the slides before haematoxylin staining. The tissues were stained in haematoxylin solution for 3–5 min, differentiated in acid alcohol, and then dipped in ammonia solution. The sections were washed in distilled water, re-hydrated in descending grades of alcohol, stained in eosin solution for 5 min and washed in distilled water for 1 min. Each mount was allowed to spread beneath the coverslip, covering all the tissues. Images were acquired using a microscope (NIKON Eclipse Ci, NIKON, Japan) and analysed with an image analysis system (NIKON Digital Sight DS-FI2, NIKON, Japan).

### Masson’s trichrome staining

All heart tissues from the area equidistant at the papillary muscle level between the ligation point and the apical section were subjected to 4% paraformaldehyde fixation and paraffin embedding. The sections were dewaxed in xylene, rehydrated in descending grades of ethanol and washed in distilled water. The sections were stained in iron haematoxylin solution for 3 min, differentiated in acid alcohol solution, washed in distilled water (kits from Beijing G-CLONE Biological Technology Co., Ltd., China, RS3960). The sections were stained in Ponceau acid fuchsin for 5–10 min and rinsed in distilled water. The slides were placed in phosphomolybdic acid solution for 1–3 min and then stained with aniline blue solution for 3–6 min. The sections were differentiated in 1% glacial acetic acid and dehydrated in ethanol, followed by xylene for 5 min. Each mount was allowed to spread beneath the coverslip by covering all the tissues. Images were acquired using a microscope (NIKON ECLIPSE E100, Japan) with an image analysis system (NIKON DS-U3, Japan). Fibrosis was analysed using Image-Pro Plus 6.0 software (Media Cybernetics, Inc., Rockville, MD, USA).

### Immunohistochemistry

Mice were euthanized and perfused with PBS or fixative. All heart tissues from the area equidistant at the papillary muscle level between the ligation point and the apical section were immersion-fixed in 4% paraformaldehyde. Tissues were trimmed, embedded, sectioned and stained for TNFα (1:200, sc-52746, Santa Cruz Biotechnology, USA). Goat polyclonal secondary antibody to rabbit IgG (H&L) was purchased from BioVision (1:1000, 6927-100, California, USA).

### Western blotting

Protein samples were extracted from the area equidistant at the papillary muscle level between the ligation point and the apical section in cold RIPA lysis buffer (MB-030–0050, Multi-Sciences Biotech, Hangzhou, China) containing a complete protease inhibitor cocktail (11697498001, ROCHE, Switzerland) and phosphatase inhibitor (4906837001, ROCHE, Switzerland). Protein samples were separated by SDS–polyacrylamide gel electrophoresis and transferred to a 0.22 μm PVDF membrane (PI88520, Millipore, USA), which was detected using specific primary antibodies (anti-TH, 1:1000, 2792, Cell Signaling Technology, USA, anti-p-TH, 1:1000, 2791, Cell Signaling Technology, USA, anti-ACHE, 1:1000, PA5-95250, Invitrogen, USA). Bound antibodies were detected using rabbit peroxidase-conjugated secondary antibody and visualized by enhanced chemiluminescence (RK-18-8816-31, Multi-Sciences Biotech, China) in a chemiluminescence imaging system (Chemi Scope 6100, Clinx Science Instruments, China). The band intensity was quantified by using Image J (National Institutes of Health, USA).

### RNA-seq and computational analysis for RNA-seq data

Extracted RNA from the area equidistant at the papillary muscle level between the ligation point and the apical section was qualified by using an Agilent 2100 Bioanalyzer (Agilent, 1309, Agilent Technologies, Inc. CA, USA) according to the manufacturer’s protocols. The RNA library was prepared according to the TruSeq RNA Sample Preparation v2 (Illumina, 15025062) protocol, followed by cluster generation and sequencing using a cBot Multiplex rehybridization plate and TruSeq SBS kit V3 (Illumina, 15021668). Sequencing was performed using an Illumina HiSeq 2000 (Illumina, USA). Data analysis was performed as previously described [22]. Before read mapping, clean reads were obtained from the raw reads by removing the adaptor sequences, reads with > 5% ambiguous bases (noted as N) and low-quality reads containing more than 20% of bases with qualities of < 20. The clean reads were then aligned to the mouse genome (version: mm10 NCBI) using hisat2 [23]. We applied the EBSeq algorithm to filter the differentially expressed genes [24] after the significance analysis, *P* value and FDR analysis under the following criteria [25]. mRNA under the following criteria: i) fold change > 2 or < 0.5 and ii) FDR < 0.05. The gene functional annotation and pathways were analysed using DAVID Bioinformatics Resources.

### qPCR

Total RNA was isolated from the area equidistant at the papillary muscle level between the ligation point and the apical section using a Fast Pure Cell/Tissue Total RNA Isolation Kit (RC101-01, Vazyme, Nanjing, China), and the concentration of isolated RNA was determined with a Qubit RNA BR assay (Invitrogen, Q10211, California, USA). Then, cDNA was prepared using HiScript Q RT Super Mix for qPCR (+ gDNA wiper) (R123-01, Vazyme, Nanjing, China) according to the manufacturer’s instructions. The mRNA levels were assessed on an ABI QuantStudio6 Q6 Real-time PCR system (ABI, USA) by qPCR using ChamQ Universal SYBR qPCR Master Mix (Q711-02, Vazyme, Nanjing, China). The relative expression of mRNA was calculated by △△Ct according to standard methods (The primer sequences were as follows: Cd84, forward: 5′-TTCCTCAGTGCAGCTTTCT-3′, reverse: 5′-CCTTGTGTCCTTCGTGGT-3′, Cd180, forward: 5′-GCAAGCCACTAATCTGAGC-3′, reverse: 5′-GTCCCCAGCCAAAGAGA-3′, Ccr2, forward: 5′-AAGGGTCACAGGATTAGGAAG-3′, reverse: 5′-ATGGTTCAGTCACGGCATA-3′, Ccr5, forward: 5′-GTGCCTGACTGCCAACA-3′, reverse: 5′-GAGACTACCTTCCCGGCTA-3′, Cx3cr, forward: 5′-TGTGCGGTCATCCTGTC-3′, reverse: 5′-CATCTCCCTCGCTTGTGT-3′, Tnf-α, forward: 5′-CGCTGAGGTCAATCTGC-3′, reverse: 5′-GGCTGGGTAGAGAATGGA-3′, Il6, forward: 5′-GCCTTCTTGGGACTGATGCT-3′, reverse: 5′-TGCCATTGCACAACTCTTTTC-3′, Scn5a, forward: 5′-GGAGGGTTGTGGTTCCTGT-3′, reverse: 5′-GTCCCTGCGGCCTATGT-3′, KChIP2, forward: 5′-AATCCCGGCAGCGCCTA-3′, reverse: 5′-CCCGGCGCTCACACA-3′, Kcne1, forward: 5′-ACATACCACACAGCAAGGGG-3′, reverse: 5′-CGATGTACTGGTGGTACGGG-3′, Cyp11b2, forward: 5′-TGAACTGAAGACAGGAGGATGG-3′, reverse: 5′-GGTATGGCTTCAAAGGGCTG-3′.)

### Statistical analysis

All data are presented as the mean ± SD. Statistical analysis was performed using one-way analysis of variance (ANOVA) with Tukey’s test using GraphPad Prism 7.0 (GraphPad Software, Inc., CA, USA) and SPSS 20.0 software (IBM, Chicago, USA). A value of *P* < 0.05 was considered to be statistically significant.

## Results

### 28-day acupuncture treatment at PC6 by acupuncture reduced susceptibility to spontaneous PVCs

**Fig. 2:**
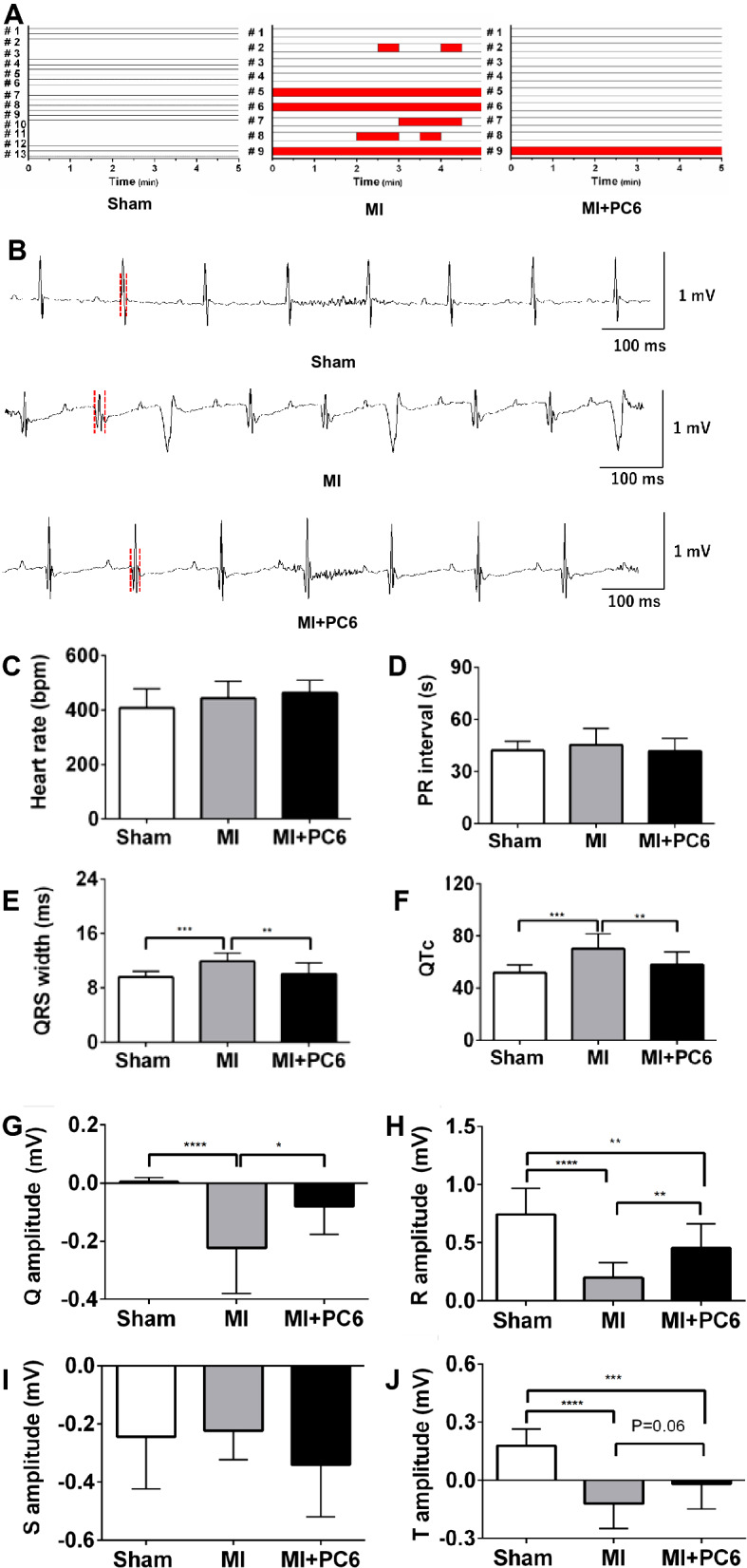
Spontaneous premature ventricular complex (PVCs) and electrophysiology in the Sham, MI and MI + PC6 groups. **A** Summary of PVCs in the Sham, MI and MI + PC6 groups. Each column indicates the onset of PVCs in each mouse. The red column represents the occurrence of a PVC every 30 s. **B** Representative normal electrocardiogram recordings in the Sham (upper panel) and MI + PC6 (bottom panel) groups. Typical traces of PVCs in the MI group (middle panel). **C**–**J** Summary of the electrophysiology, including the heart rate, PR interval, QRS width, QTc, Q amplitude, R amplitude, and S amplitude and T amplitude in the Sham, MI and MI + PC6 groups. *p < 0.05, **p < 0.01, ***p < 0.001, ****p < 0.0001

After 4 weeks of acupuncture intervention, spontaneous PVCs was not observed in the Sham group (0%), emerged in the MI group (67%), and decreased to 11% in the MI + PC6 group. The typical electrocardiogram traces and the incidences of PVCs are shown in Fig. 2A and B. Compared with those in the Sham group, the QRS width and QTc were significantly increased in the MI group. Acupuncture shortened the QRS width and QTc in the MI + PC6 group in comparison with the MI group. In addition, the R wave amplitude was also reduced in the MI group, whereas it was significantly reversed after the intervention. Spontaneously, a significant deep pathological Q wave was observed in the MI group, but it was decreased in the acupuncture group. Moreover, abnormally deep inverted T waves in the MI group were significantly lowered in the mice receiving acupuncture (Fig. 2C–J).

### Acupuncture improved systolic function and reduced fibrosis

**Fig. 3:**
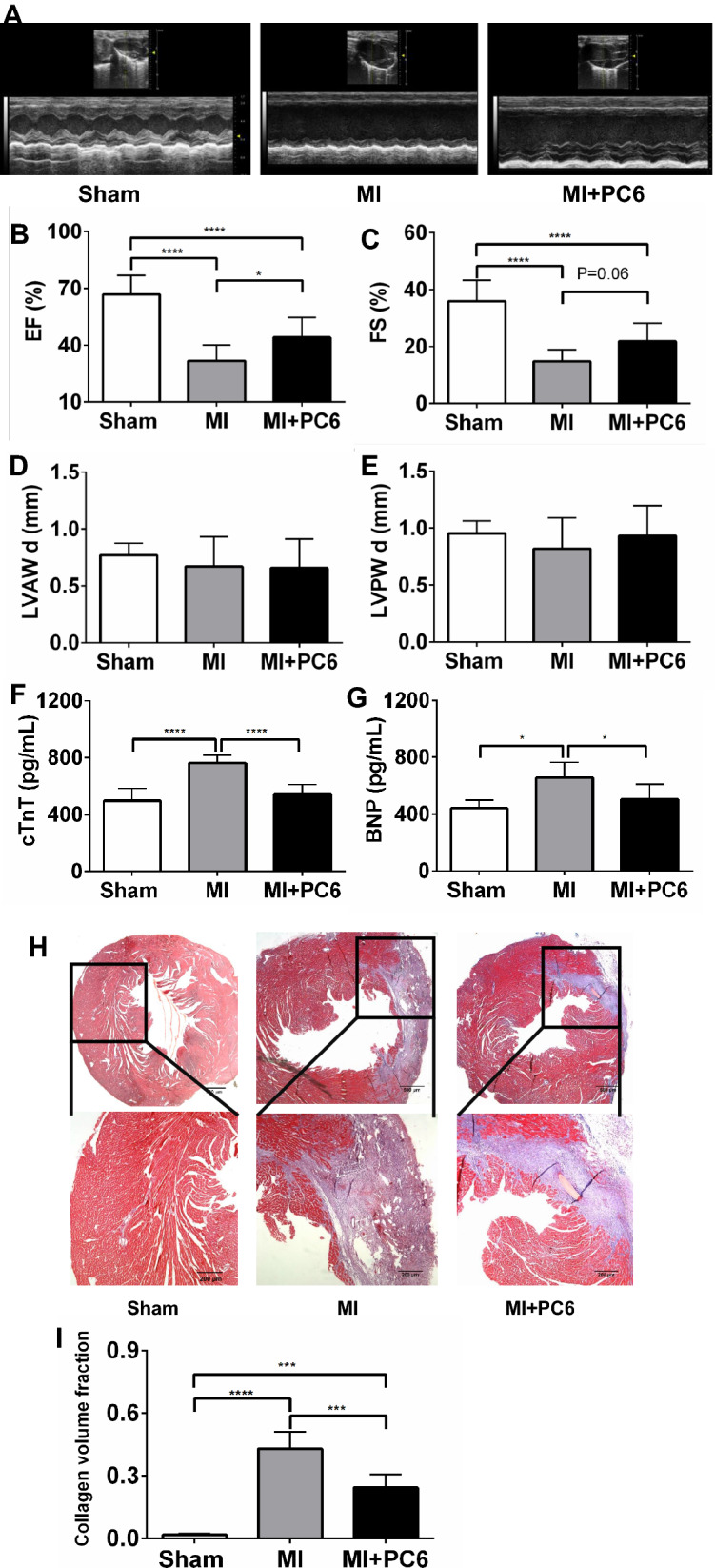
Acupuncture improved cardiac function and reduced fibrosis. **A** Representative echocardiograms from parasternal long axis images in the Sham, MI and MI + PC6 groups. A summary of echocardiographic parameters is listed below (**B**–**E**). **B** Ejection fraction (EF), **C** fractional shortening (FS), **D** left ventricular anterior wall in diastole (LVAWd), **E** left ventricular posterior wall in diastole (LVPWd), **F** the serum cTnT level in the Sham, MI and MI + PC6 groups **G** the serum BNP level in the Sham, MI and MI + PC6 groups. **H** Tissue sections and fibrosis in the ventricle determined by calculating collagen deposition after Masson's trichrome staining in the Sham (left), MI (middle) and MI + PC6 (right) group mice. Black bar: 200 μm. ****p < 0.0001

Typical echocardiogram traces for the effects of acupuncture on cardiac function and structure are shown in Fig. 3A. Both EF and FS in the MI group were significantly reduced compared with those in the Sham group, but the acupuncture intervention slightly reversed the reduction in EF and FS (*p* = 0.06) (Fig. 3B and C). No significant difference was detected at the diastolic end of the left ventricular posterior and anterior wall thickness (LVPWd and LVAWd) among these 3 groups (Fig. 3D and E). Serum cTnT and BNP levels increased after MI, whereas acupuncture lowered these parameters (Fig. 3F and G). Masson’s trichrome staining of the ventricles showed that acupuncture at PC6 significantly decreased the fibrosis levels in the ventricle, which appeared in the infarct area in the MI group (Fig. 3H and I).

### Acupuncture altered the expression of genes related to the inflammatory response

**Fig. 4:**
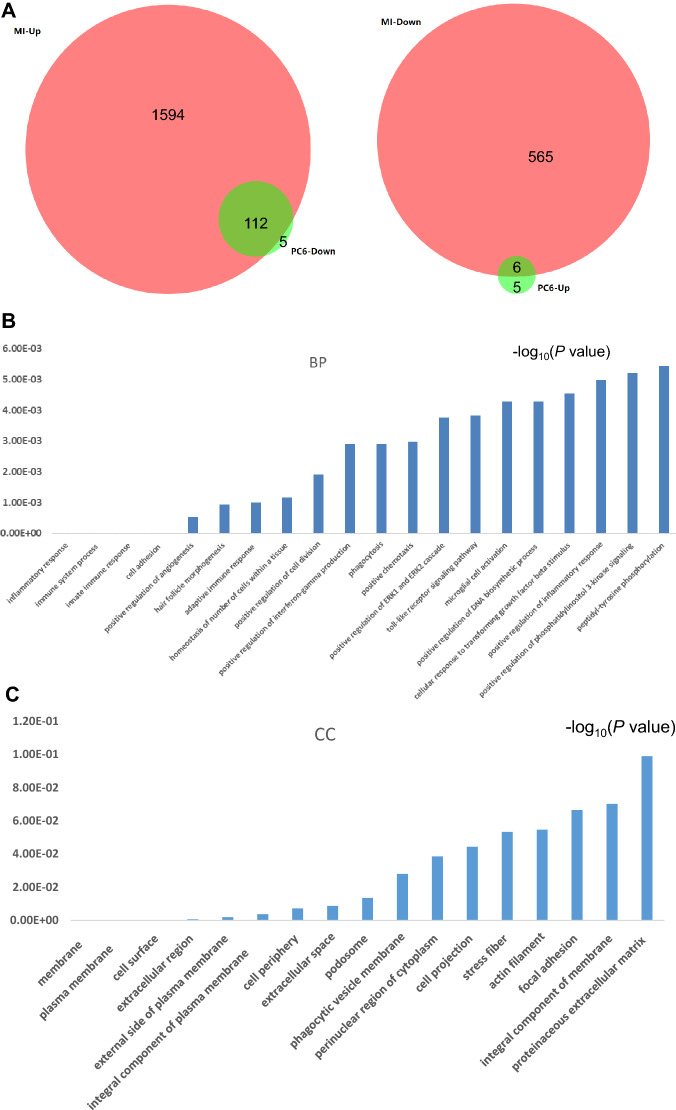

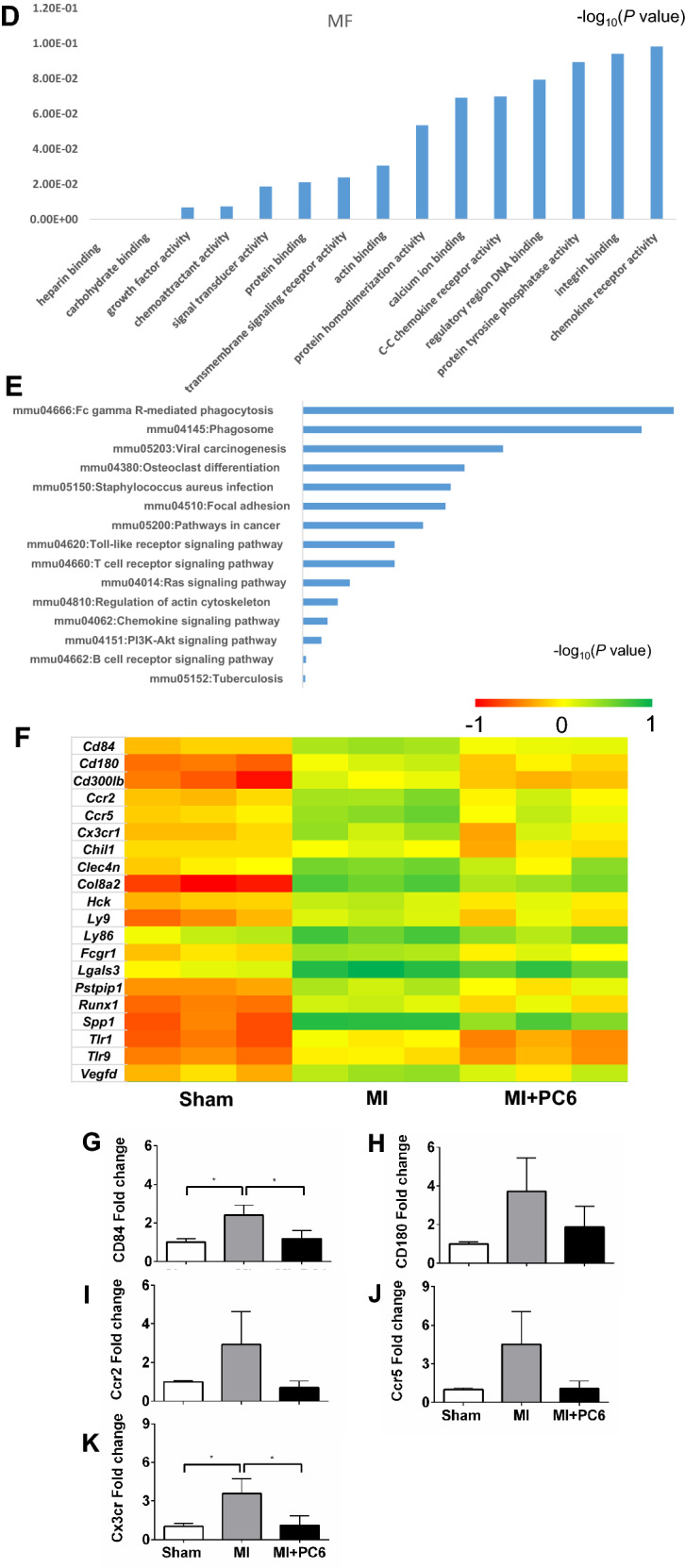
Differentially expressed genes (DEGs) altered by acupuncture treatment (n = 3 per group). **A** Venn diagram of the DEGs. **B**–**D** GO analysis of the biological processes, cell components and molecular functions. **E** KEGG pathways of 112 coregulated genes. **F** Heatmap of the top 20 genes among 112 coregulated genes between the MI and MI + PC6 groups. **G**–**K** qPCR verification of some inflammation-related genes among the 112 coregulated genes. For qPCR data are collected at least from two independent experiments carried out in triplicate

To investigate the underlying molecular mechanisms by which 28 days of acupuncture treatment at PC6 protected against MI injury and post-MI PVCs, we extracted RNA from heart tissues and conducted gene expression profiling for the three groups by using next-generation high-throughput sequencing (RNA-seq analysis). Our results showed that 2277 genes were differentially expressed in the MI group compared to the Sham group. Of these 2277 genes, 1706 (74.9%) genes were upregulated, and 571 (25.1%) genes were downregulated. Acupuncture at PC6 for 28 days downregulated 117 (91.4%) genes and upregulated 11 (8.6%) genes compared with those in the MI group (Table 1). Venn diagrams showed that of the 1706 upregulated genes from the MI group, 6.6% (112) were downregulated by acupuncture, and of the 571 downregulated genes from the MI group, 1% (6) were upregulated by acupuncture (Fig. 4A). Gene ontology (GO) annotation indicated that the coregulated genes in the heart were mainly expressed in biological processes of the inflammatory response, immune system processes, innate immune responses, cell adhesion, positive regulation of angiogenesis, adaptive immune responses, positive regulation of cell division, and positive regulation of interferon-gamma production (Fig. 4B–D). Kyoto Encyclopedia of Genes and Genomes (KEGG) pathway analysis from DAVID confirmed that these genes belonged to many functional pathways that were involved in the B cell receptor signalling pathway, PI3K-Akt signalling, Ras signalling, Fc gamma R-mediated phagocytosis, phagosome, toll-like receptor signalling, and focal adhesion (Fig. 4E). Heatmaps were created with the top 20 pathologically coregulated genes (including *Cd*84*, Cd*180*, Ccr*2*, Ccr*5*, Cx3cr*1*, Col8a*2*, Runx*1*, Vegfd*) between the MI and acupuncture groups. In Fig. 4F, the red colour indicates genes with higher expression, and the green colour indicates genes with lower expression.

**Table 1:** Differentially expressed genes (DEGs) with a log2(FC) >|± 1| and *p* value < 0.05

DEGs	MI vs. Sham	MI + PC6 vs. MI
Up-regulated	1706 (74.9%)	11 (8.6%)
Down-regulated	571 (25.1%)	117 (91.4%)
Total	2277 (100%)	128 (100%)

### Acupuncture alleviated the inflammatory response induced by MI in both the heart and serum

**Fig. 5:**
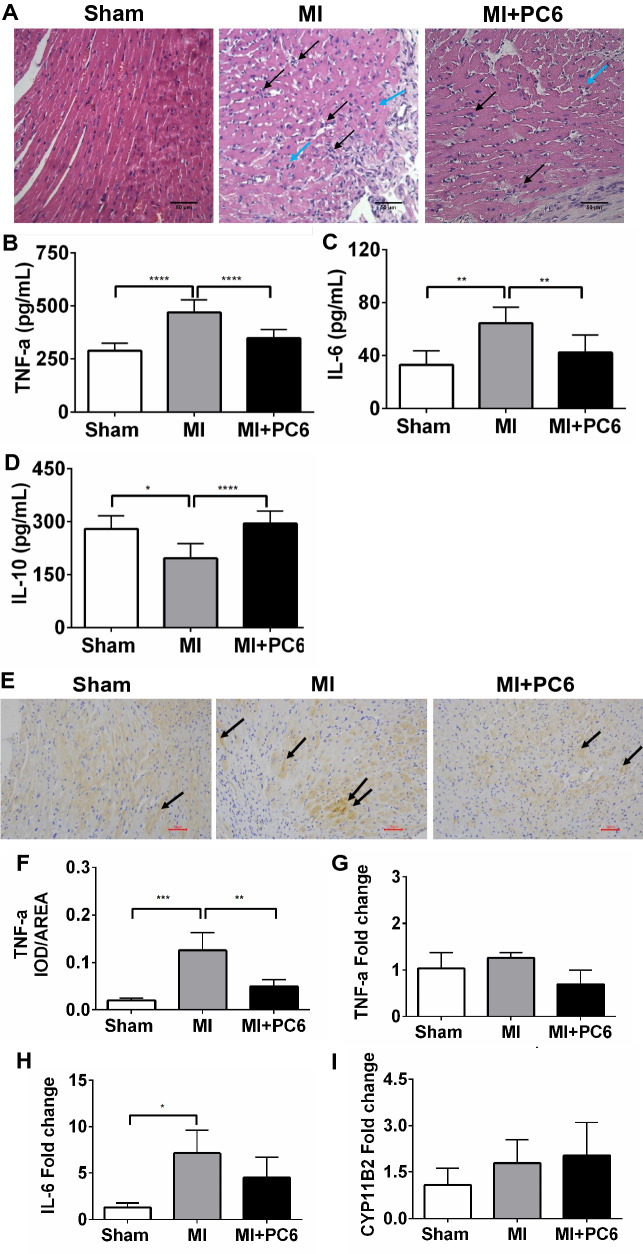
Acupuncture alleviated the inflammatory response induced by MI. **A** Haematoxylin and eosin staining (HE) staining of heart tissue in each group (black arrow indicates inflammatory cells, and blue arrow indicates foam cells, n = 3 per group). **B**–**D** Plasma levels of TNF-α, IL-6, and IL-10 in the Sham, MI and MI + PC6 groups. **E**, **F** Representative TNF-α immunohistochemical staining and analysis of heart tissues in Sham (n = 3, left panel), MI (n = 3, middle panel), and MI + PC6 (n = 3, right panel) mice. (G-I) *Tnf*-α, *Il*-6 and *Cyp11b*2 mRNA expression in the heart

Since most of the DEGs were primarily enriched in the inflammatory response signalling pathway, we verified some inflammation parameters by qPCR, haematoxylin and eosin (HE) staining, ELISA and immunohistochemistry. qPCR confirmed some DEGs, including *Cd*84, *Cd*180, *Ccr*2, *Ccr*5, and *Cx3cr*1, which are involved in the inflammatory response. The results showed a similar tendency, as shown by RNA-Seq (Fig. 4G–K). HE staining detected inflammatory cell infiltration and foam cells in the atria and ventricles in the MI group, which were rarely observed in the Sham and acupuncture groups (Fig. 5A). The plasma levels of TNF-α and IL-6 were significantly increased in the MI group compared with those in the Sham group but were decreased in the acupuncture group (Fig. 5B and C). IL-10 was reduced in the MI group compared with that in the Sham group, but acupuncture intervention significantly increased the IL-10 level (Fig. 5D). Immunohistochemistry also indicated that acupuncture reduced TNF-α expression, which was elevated in MI cardiac tissue **(**Fig. 5E and F). Interleukin-6 (*Il*-6) mRNA expression also showed a similar change tendency, while *Tnf*-α and *Cyp11b*2 mRNA expression displayed no significant change (Fig. 5G–I). This could be due to the difference in expression timing between the protein and mRNA levels.

### Acupuncture modulated enhanced sympathetic activation and renin release

**Fig. 6:**
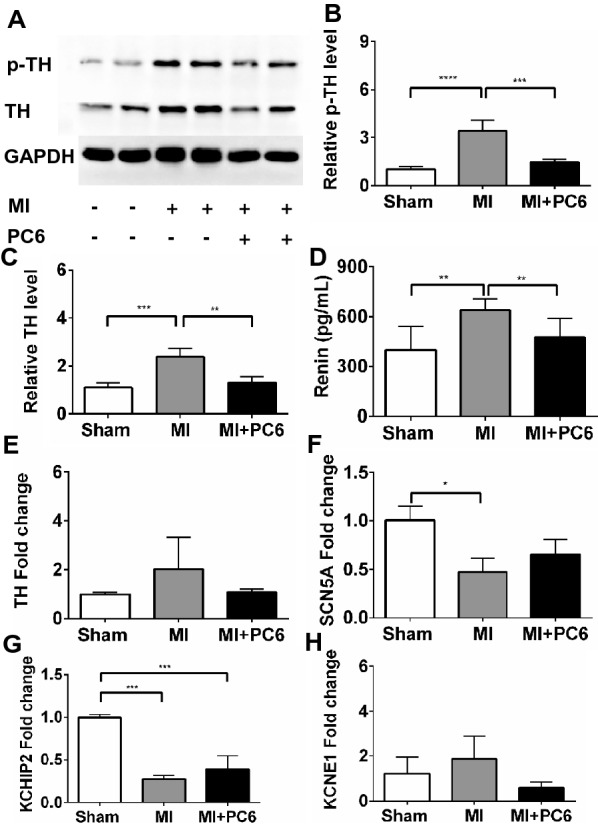
Acupuncture decreased cardiac sympathetic hyperactivity and renin release. **A**–**C** Western blot analysis and quantification of phosphonate tyrosine hydroxyzine (p-TH) and tyrosine hydroxyzine (TH) protein levels (n = 3 per group). **D** Plasma level of renin in the Sham, MI and MI + PC6 groups. **E**–**H** *Th*, *Scn*5A, *KChIP*2, and *Kcne*2 mRNA levels in cardiac tissue among the three groups

Western blot analysis showed that tyrosine hydroxylase (TH) was upregulated in the ventricle tissue 4 weeks after MI, but this upregulation was reversed by acupuncture intervention (Fig. 6A–C, E). ELISA verified that the renin level in the serum was also increased in the MI group, whereas it was reduced after acupuncture treatment (Fig. 6D). These results suggested that 28 days of acupuncture intervention suppressed sympathetic hyperactivity and activation of the RAAS. Since neurohormones modulated ion channel function and expression, which led to arrhythmia, the transcript levels of *Scn*5A, *KChIP*2 and *Kcne*1 were detected by qPCR, and the results showed that acupuncture tended to increase *Scn*5A and *KChIP*2 but decrease *Kcne*1 expression levels (Fig. 6F–H).

## Discussion

Arrhythmia is one of the most common complications after myocardial infarction. However, the safe and effective treatment strategies are not established yet and about 50% of patients die from ventricular arrhythmia after myocardial infarction [26]. Clinical observations and animal research have reported that acupuncture can alleviate several kinds of arrhythmias [27, 28], but vague mechanism study limited its application and popularization. In this study, we observed the protective effect of 28-day treatment with acupuncture at PC6 on ventricular arrhythmia in a MI mice model, and found that the incidence of spontaneous PVCs was reduced, and the systolic function was improved by acupuncture intervention. Our results provided scientific experimental evidence that acupuncture can reduce post-MI of PVCs, which would be beneficial for its application and promotion clinically.

The underlying pathophysiology of PVCs includes reentry, enhanced automaticity, and triggered activity [29]. Reentry occurs when an area of one-way block in the Purkinje fibres and a second area of slow conduction are present. This condition is frequently seen in patients after MI and creates areas of differential conduction and recovery due to myocardial scarring or ischaemia [30]. Cardiac fibrosis is a crucial determinant of myocardial heterogeneity and provides the substrate for reentry arrhythmias [31]. In this study, MI induced arrhythmias and fibrosis, but acupuncture treatment at PC6 significantly prohibited these pathological processes, indicating that the reduced arrhythmias could be attributed to the decrease in the substrate for arrhythmias and fibrosis and that the decreased fibrosis further improved the systolic function of the post-MI heart.

An intense inflammatory response occurs rapidly after myocardial infarction, which is not only essential for cardiac repair, but also is implicated in the pathogenies of post-infarction remodelling and heart failure [6]. Our RNA-seq data confirmed that numerous co-regulated genes were involved in the inflammatory response, immune system process, innate immune response, cell adhesion, and apoptosis pathways (Fig. 4B). Inflammation and fibrosis play an important role in the development and progression of arrhythmia, resulting in reentry and triggers of arrhythmia [32]. In the MI + PC6 group mice, we observed that acupuncture reduced fibrosis and the inflammatory response, suggesting that the anti-arrhythmic effect may result from the inhibition of inflammation. Actually, the upregulation and production of cytokines, such as TNF-α and IL-6, represent an intrinsic or an innate stress response against myocardial injury [33], and they were regulated by acupuncture [34, 35]. In the present study, 28-day acupuncture intervention lowered serum and cardiac TNF-α levels and serum IL-6 levels. The elevation in cytokine expression preceded the consequent increase in local matrix metalloproteinase activity in the infarct area, as well as the increase in natriuretic peptides (ANP and BNP) and collagen expression in the non-infarcted myocardium [36]. Consistently, we found that BNP secretion from heart tissue was increased after MI injury and reduced by acupuncture, accompanied by a change in fibrosis.

Abnormal automaticity is one of the most important underlying electrophysiologic mechanisms for arrhythmias under MI and post-MI conditions [37]. When MI occurs, cardiac injury signals are “sent” to stellate ganglia and result in increased cardiac sympathetic innervation and activation of the renin-angiotensin aldosterone system, which plays an essential role in the genesis and maintenance of ventricular arrhythmias, not only in the acute phase of myocardial ischaemia but also in the post-infarction remodelling process [38, 39]. In this study, TH and renin were increased after MI injury, indicating increased cardiac sympathetic innervation and activation of the renin-angiotensin aldosterone system. 28-day intervention with acupuncture inhibited the transcriptome level of tyrosine hydroxylase and the protein levels of tyrosine hydroxylase and phosphorylated tyrosine hydroxylase in the border zone of MI hearts. In addition, it also reduced the release of serum renin levels. It is known that renin is produced and secreted by kidneys, controls the activation of the hormone angiotensin cascade and stimulates the adrenal glands to produce aldosterone. Many studies have demonstrated that sustained activation of the sympathetic system and RAAS remodelled-ion channels are very important regulators of the cardiac action potential [40, 41]. In this study, we also detected that the transcriptome levels of *Scn*5A (I_Na_) and *KChIP*2 (I_to_) were downregulated but that *Kcne*1 (I_Ks_) was upregulated in MI group mice. Acupuncture treatment slightly upregulated *Scn*5A (I_Na_) and *KChIP*2 (I_to_) but downregulated K*cne*1 (I_Ks_) compared to the corresponding expression in the MI group. The action potential in ventricular cardiac muscle results from coordinated activation and deactivation of ion channels [42], which begins with a rapid upstroke caused by I_Na_. Downregulation of S*cn*5A (I_Na_) resulted in slow conduction and a wide QRS after MI. I_to_ is the major current contributing to repolarization. Downregulation of I_to_ leads to a delay in phase 3 repolarization of the action potential and a long QT after MI in mice. I_Ks_ are also critical for the repolarization phase of the cardiac action potential. Upregulation of *Kcne*1 (I_Ks_) indicated sympathetic hyperactivity. Although the regulatory role in each ion channel is mild, balancing these ion channels by acupuncture intervention may partly contribute to the lower incidence of PVCs after MI. The changes in QRS and QT interval also support this hypothesis.

In this study, we proved that acupuncture at Neiguan for 28 days can suppress PVCs occurring post-myocardial infarction by alleviating inflammation and fibrosis. Some autonomic function-related genes and proteins were detected at the same time. Regarding the detailed mechanisms by how does acupuncture reduce inflammation and regulate autonomic function, more experimental verifications are needed in the future.

## Conclusion

**Fig. 7:**
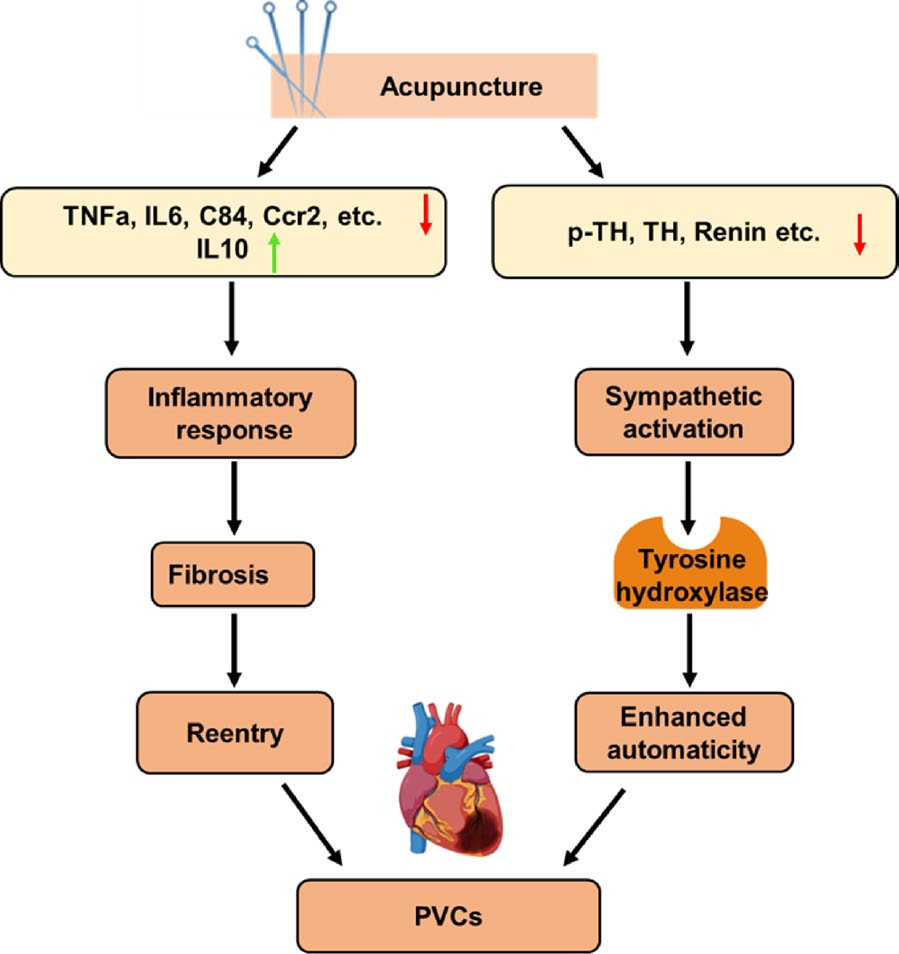
Graphical abstract of the mechanism for the protective effect of acupuncture on myocardial infarction-induced arrhythmia. Here we demonstrate the two main pathophysiology cause of PVCs (reentry and enhanced automaticity), which 28-day acupuncture may exert beneficial adjustment on the ischemic myocardium (red arrow indicates inhibition, while green arrow represents improvement)

In conclusion, the current study revealed that repeated acupuncture intervention at PC6 reduced spontaneous PVCs and improved systolic function, probably by suppressing inflammatory response-mediated fibrosis, reducing the substrate for reentry, and inhibiting sympathetic hyperactivity. Our findings provide experimental evidence for the efficacy of acupuncture treatment in delaying cardiac remodelling and spontaneous ventricular arrhythmia (Fig. 7).

## Data Availability

The datasets used and/or analysed during the current study are available from the corresponding author on reasonable request.

## Abbreviations

MI: Myocardial infarction
PVC: Premature ventricular complex
LAD: Left anterior descending
ECG: Electrocardiogram
EF: Ejection fraction
FS: Fractional shortening
LVPWd: Left ventricle posterior wall thickness at diastole
LVAWd: Left ventricle anterior wall thickness at diastole
cTnT: Cardiac troponin T
BNP: Brain natriuretic peptide
DEGs: Differentially expressed genes
GO: Gene Ontology
KEGG: Kyoto Encyclopedia of Genes and Genomes
Ccr2: CC Chemokine Receptor 2
Ccr5: CC Chemokine Receptor 5
Cx3cr: C-X3-C Motif Chemokine receptor 1
IL-6: Interleukin-6
IL-10: Interleukin-10
TNF-α: Tumour necrosis factor-α
Cyp11b2: Cytochrome P450 Family 11 Subfamily B Member 2
KChIP2: Potassium Voltage-Gated Channel Interacting Protein 2
Scn5A: Sodium Voltage-Gated Channel Alpha Subunit 5
Kcne1: Potassium Voltage-Gated Channel Subfamily E Regulatory Subunit 1
TH: Tyrosine hydroxylase
